# The Effect of Exogenous Cadmium and Zinc Applications on Cadmium, Zinc and Essential Mineral Bioaccessibility in Three Lines of Rice That Differ in Grain Cadmium Accumulation

**DOI:** 10.3390/foods12214026

**Published:** 2023-11-04

**Authors:** Michael Tavarez, Michael A. Grusak, Renuka P. Sankaran

**Affiliations:** 1 The Graduate Center, City University of New York, New York, NY 10016, USA; michael.tavarez@lehman.cuny.edu; 2 Department of Biological Sciences, Lehman College, City University of New York Bronx, New York, NY 10468, USA; 3 USDA-ARS Edward T. Schafer Agricultural Research Center, Fargo, ND 58102, USA; mike.grusak@usda.gov

**Keywords:** cadmium, zinc, rice, bioaccessibility, dietary exposure, nutrition

## Abstract

Millions of people around the world rely on rice (*Oryza sativa*) for a significant portion of daily calories, but rice is a relatively poor source of essential micronutrients like iron and zinc. Rice has been shown to accumulate alarmingly high concentrations of toxic elements, such as cadmium. Cadmium in foods can lead to renal failure, bone mineral density loss, cancer, and significant neurotoxicological effects. Several strategies to limit cadmium and increase micronutrient density in staple food crops like rice have been explored, but even when cadmium concentrations are reduced by a management strategy, total cadmium levels in rice grain are an unreliable means of estimating human health risk because only a fraction of the minerals in grains are bioaccessible. The goal of this work was to assess the influence of cadmium and zinc supplied to plant roots on the bioaccessibility of cadmium and essential minerals from grains of three rice lines (GSOR 310546/low grain Cd, GSOR 311667/medium grain Cd, and GSOR 310428/high grain Cd) that differed in grain cadmium accumulation. Treatments consisted of 0 μM Cd + 2 μM Zn (c0z2), 1 μM Cd + 2 μM Zn (c1z2), or 1 μM Cd + 10 μM Zn (c1z10). Our results revealed that an increased grain cadmium concentration does not always correlate with increased cadmium bioaccessibility. Among the three rice lines tested, Cd bioaccessibility increased from 2.5% in grains from the c1z2 treatment to 17.7% in grains from the c1z10 treatment. Furthermore, Cd bioccessibility in the low-Cd-accumulating line was significantly higher than the high line in c1z10 treatment. Zinc bioaccessibility increased in the high-cadmium-accumulating line when cadmium was elevated in grains, and in the low-cadmium line when both cadmium and zinc were increased in the rice grains. Our results showed that both exogenous cadmium and elevated zinc treatments increased the bioaccessibility of other minerals from grains of the low- or high-grain cadmium lines of rice. Differences in mineral bioaccessibility were dependent on rice line. Calculations also showed that increased cadmium bioaccessibility correlated with increased risk of dietary exposure to consumers. Furthermore, our results suggest that zinc fertilization increased dietary exposure to cadmium in both high and low lines. This information can inform future experiments to analyze genotypic effects of mineral bioavailability from rice, with the goal of reducing cadmium absorption while simultaneously increasing zinc absorption from rice grains.

## 1. Introduction

Cadmium (Cd) is one of the most toxic transition metals [1]. The toxin can be found at trace levels of about 0.2 mg/kg in soils, but its ubiquity continues to increase due to industrialization [2,3]. Anthropogenic activities like sewage treatment, pigment production, and agriculture are amongst the greatest Cd sources, contributing an estimated 8000 to 10,000 metric tons of Cd to the environment every year [2,3,4,5,6,7]. Cadmium in soils is of special concern because plants can accumulate high concentrations of Cd in edible tissues, which can then be transferred through the food chain leading to renal failure, bone mineral density loss, and cancer [8,9,10,11,12,13,14,15,16,17]. An estimated 98% of Cd in humans comes from dietary intake, with estimates of 70 to 80% coming from plant-based foods [7]. Average U.S. Cd consumption is about 0.54 μg/kg body weight/week, which is about a fifth of the tolerable weekly intake of 2.5 μg/kg body weight/week [18]. Daily intake in regions with highly contaminated soils have been recorded as high as 18.6 μg/kg/day due largely to its prevalence in plant foods [9,19,20].

The potential for adverse environmental health exposure to this toxic metal is significant because grains contribute up to 27% of the human dietary consumption of Cd [21]. Several studies have found most grain containing baby foods contain some level of Cd, which is due to the presence of Cd in many soils used for grain production around the world [22,23,24,25]. Limitations of 0.2 mg/kg Cd in polished rice grain have been set by the United States Department of Agriculture (USDA), European Union, and China National Food and Safety Standards to try and limit the toxin from entering the food chain [26,27,28]. Several techniques including plant breeding, genetic engineering, and agronomic practices, such as the use of zinc fertilizers or chloride-based irrigation techniques, are currently being explored as a means of reducing grain Cd and thereby limiting Cd consumption [20,29,30,31,32,33,34,35,36,37].

Dietary exposure to Cd depends not only on the concentration of Cd in the food but also on the bioavailable fraction of Cd from the food [38]. Metal–micronutrient interactions further influence how toxic metals and beneficial micronutrients such as zinc are taken up and transported to plant tissues [39]. Both bioavailability (the amount of a nutrient that reaches systemic circulation) and bioaccessibility (BA) (the amount of a nutrient that is available for absorption after digestion) are also influenced by other factors including post agronomic processing, food preparation (i.e., boiling vs. pressure cooking), plant genotype, and mineral interactions [40,41,42,43,44]. Several rice genotypes have been found to differ significantly in Cd and Zn grain concentrations due to varying mechanisms of uptake and sequestration [45,46,47]. In this study, we used three rice lines (GSOR 310546, GSOR 311667, and GSOR 310428) that differ in grain Cd and Zn concentrations from the Genetic Stocks Oryza (GSOR) Mini Core Collection. Furthermore, evidence suggests interactions between minerals such as Cd and Zn in growing media can affect minerals such as Ca, Cu, Fe, Mg, Mn, Ni, and P in plant foods, which must be taken into consideration when assessing health risk [48,49,50].

Comprehensive understanding of rice genotype and mineral interactions influencing bioaccessibility are important to accurately predict risks from consuming Cd-contaminated rice and rice products. The main goals of this work were to analyze the influence of Cd and Zn interactions on the bioaccessibility of Cd and other minerals in different lines of rice and to assess the risks associated with consuming these contaminated plant foods. Our results provide information that can assist in efforts to produce rice grains with enhanced nutritional quality while simultaneously reducing the bioavailability of Cd.

## 2. Materials and Methods

### 2.1. Plant Growth

Rice (*Oryza sativa*) accessions 310546 (546), 310667 (667), and 310428 (428) (Table 1) were selected from the USDA-ARS, Dale Bumpers National Rice Research Center (Stuttgart, Arkansas, Genetic Stocks Oryza Collection-GSOR) to represent low-, medium-, and high-grain Cd accumulators, respectively [45]. When grown hydroponically in the presence of 1 μM Cd, rice lines 546, 667, and 428 accumulated 0.3, 1.09, and 2.0 μg/g dry weight (DW), respectively [39]. Seeds were surface-sterilized in a 1% (*v*/*v*) sodium hypochlorite solution, imbibed overnight in 18 MΩ H_2_O, and germinated in 18 MΩ H_2_O-moistened-filter-paper-lined Petri dishes for 5 days. Petri dishes were wrapped in parafilm and kept in the dark. Healthy seedlings were transplanted to a 4 L hydroponic system filled with a modified Johnson’s nutrient solution. The nutrient solution was continuously aerated and replaced weekly with 2 mM KNO_3_, 1 mM Ca (NO_3_)_2_, 1 mM KH_2_PO_4_, 1 mM MgSO_4_, 25 μM CaCl_2_, 25 μM H_3_BO_3_, 0.5 μM H_2_MoO_4_, 0.1 μM NiSO_4_, 2 μM MnSO_4_, 0.5 μM CuSO_4_, iron supplied as 20 μM Fe (III) HEDTA (N-(2-hydroxymethy) ethylenediaminetriacetic acid), and 2 mM 2-(N-morpholino) ethane–sulfonic acid (MES; Sigma Chemical, St. Louis, MO, USA) to buffer the solution to pH 6.0. The experiment consisted of a total of 18 plants grown in 9 pots for each of the three lines. There were 2 plants per pot in 3 pots total for every line × treatment permutation. All pots were continuously aerated. Plants were maintained in natural lighting plus supplemental metal halide lamps [100 W], allowing for a 16/8 h photoperiod at 25 ± 3 °C day/23 ± 3 °C night temperatures in the Lehman College (Bronx, NY) Science greenhouse.

A completely randomized factorial design was used to designate the pots and allow replicates (*n* = 3) for the three lines (546, 667, and 428) and treatment. When plants grew to the 4-to-5-leaf stage, a fresh solution was given where treatments were imposed in conjunction with nutrient solutions. Treatments consisted of a combination of different Cd and Zn concentrations: c0z2, c1z2, and c1z10 were made up of 0 μM Cd + 2 μM Zn (c0z2), 1 μM Cd + 2 μM Zn (c1z2), or 1 μM Cd + 10 μM Zn (c1z10) using CdCl_2_ and ZnSO_4_. At full maturity, panicles were harvested and then threshed and dehusked manually. Dehusked brown rice are referred to as grains for the remainder of this experiment. Grains were then separated in half to be used for mineral analysis and bioaccessibility experiments.

### 2.2. Physiologically Based Extraction Test

Bioaccessibility (BA) was determined in uncooked rice grains. All assays were performed in triplicates. Rice grains from each line and treatment were crushed by mortar and pestle. Rice flour from crushed samples and NIST (National Institute of Standards and Technology) standard reference material 1568b was then put through a modified in vitro physiologically based extraction test (PBET) [51,52] to estimate minerals accessible to the lumen of the human gastrointestinal tract. All assay solutions were prepared fresh on the day of the experiment in accordance with procedures from the study of Narayanan et al., 2019, with slight modifications [53]. Saline, pancreatin/bile, sodium bicarbonate, and pepsin solutions were prepared shortly before use as follows: for saline, 140 mM NaCl + 5 mM KCl (Sigma, St. Louis, MO, USA) were dissolved in 18 Ω MQ H_2_O; for pancreatin/bile, 0.067 g of purified pancreatin (Fisher, Hampton, NH) + 0.3 g porcine bile extract (Sigma, St. Louis, MO) were dissolved in 64 mL of 0.1 M NaHCO_3_; for sodium bicarbonate, a 0.1 M and a 1 M NaHCO_3_ were made by dissolving 2.01 g NaHCO_3_ (Sigma, St. Louis, MO, USA) in 240 mL of 18 Ω MQ H_2_O and 5.04 g NaHCO_3_ in 60 mL of 18 Ω MQ H_2_O; and for pepsin, 0.26 g of pepsin from porcine gastric mucosa (Sigma, St. Louis, MO, USA) was dissolved in 13.33 mL of 0.1 M HCl.

For the assay, rice flour (1 g) was mixed with 6.67 mL of the 0.9% saline solution before the pH of all samples was adjusted to 2.0 using 1 N HCl. Samples were then mixed with 0.83 mL of pepsin and incubated for 1 h at 37 °C on a rocking platform at max speed. After the 1 h peptic digestion, the pH of all samples was adjusted to 5–5.5 with 1 M NaHCO_3_. All samples then received 4.17 mL of the pancreatin/bile solution before pH was increased to 7 with 1 M NaHCO_3_. Samples were then incubated at 37 °C for 2 h on a rocking platform at maximum speed, after which they were centrifuged for 10 min at 10,000 RPM before supernatants were collected. Blanks containing no flour were run with each set of samples as a negative control. To ensure degradation of organic compounds, supernatants collected were digested in concentrated nitric acid and 30% hydrogen peroxide (trace metal grade), resuspended in 1% nitric acid, and filtered using 0.45 μM syringe filters. The samples were then analyzed for essential minerals and Cd by inductively coupled plasma optical emission spectroscopy (ICP-OES) that was calibrated with certified standards. Negative-control technical blanks containing experimental solutions without rice were analyzed with each digestion. Mineral concentrations were used to calculate content in the digestible fraction. Technical blank content was subtracted from mineral content of the digestible fraction to ensure accuracy and negate inorganic minerals from digestive solutions. Mineral content (per g of rice flour) was then used to calculate bioaccessibility% using Equation (1):

Equation (1): bioaccessibility percent

(1) $$\mathrm{Bioaccessibility}\%\;(\mathrm{BA})=\frac{\mathrm{migrated}\;\mathrm{mineral}\;\mathrm{content}\;\mathrm{from}\;\mathrm{PBET}\;\mathrm{assay}\;}{\;\mathrm{grain}\;\mathrm{mineral}\;\mathrm{content}\;}\times 100$$

### 2.3. Mineral Analysis

Grains from individual plants were harvested at reproductive maturity and oven-dried at 60 °C for 48 h. Dry tissue was homogenized using a Wiley mill with a 0.2 mm pore (Thomas Scientific, Swedesboro, NJ, USA). Rice standard reference material (NIST 1568b) [52] was run in triplicate and used to validate experimental procedures. All rice flour samples (~0.25 g) were digested using a nitric acid tissue digestion protocol outlined in [11]. In short, 3 mL of concentrated nitric acid was used to predigest samples for 12 h at room temperature and then for 1 h at 100 °C in a BD50 Digestion Block (Seal Analytical, Toronto,ON, Canada). Once cooled, 4 mL of 30% hydrogen peroxide was added and temperatures were periodically increased to 250 °C over the course of 6 h to dissolve all organic material. Digests were resuspended in 10 mL of 1% nitric acid and filtered using a 0.22 μm pre syringe filters. The acids used were trace metal-grade (Fisher Scientific, Pittsburg, PA, USA) and the water used was deionized using the MilliQ system (Millipore, Billerica, MA, USA). Cadmium sulfate was used as positive controls and blanks were used for every digestion run. Samples were analyzed for nine essential minerals (Ca, Mg, P, S, K, Cu, Fe, Zn, Mn) and Cd using inductively coupled plasma optical emission spectroscopy (ICP-OES; iCAP 7000; Thermo Electron North America LLC, Madison, WI, USA) that was calibrated with certified standards to detect mineral concentrations as described previously [54]. Negative controls (blanks) were processed and analyzed to monitor for background levels of minerals in the digestions and resuspension solutions. Background mineral levels were subtracted from sample values before concentrations were calculated. Reported averages and standard error are derived from concentrations from three biological replicates (three pots of six plants).

### 2.4. Dietary Exposure

Weekly dietary exposure (DE) was calculated for men, women, and children to estimate the long term impacts of prolonged consumption of Cd contaminated rice from three cultivars that differed in grain Cd concentration. An average body mass of 70 kg, 60 kg, and 13 kg were used to represent men, women, and children, respectively. For the purpose of this experiment, an average daily consumption of 430 g, 320 g and 100 g was assumed for men, women, and children, respectively, based off a study on recommended and accepted published estimates of rice intake [55,56]. Lastly, generally accepted rice mineral absorption of 5% was used in conjunction with mineral concentrations (Table 2) and bioaccessibility calculated above to determine dietary exposure [57,58]. The dietary exposure was calculated using Equation (2):

Equation (2): dietary exposure

(2) $$\mathrm{Dietary}\;\mathrm{Exposure}\;(\mathrm{DE})=\frac{\mathrm{mass}\;\mathrm{consumed}/\mathrm{day}\times \mathrm{absorption}\%\;\text{×}\mathrm{BA}\;\text{×}\;\mathrm{mineral}\;\mathrm{concentration}\;}{\;\mathrm{body}\;\mathrm{mass}\;\text{×}\;7\;\mathrm{days}\;\mathrm{per}\;\mathrm{week}\;}$$

### 2.5. Statistical Analysis

Analysis of variance and Pearson’s correlation coefficients were performed using GraphPad Prism version 9.1 (GraphPad, San Diego, CA, USA). Two-way analysis of variance (ANOVA) multiple-comparisons test with a Bonferroni correction were used to analyze statistical differences in mean BA (*n* = 3) within a line across treatments and within a treatment across lines (*p* ≤ 0.05). Significant differences between lines within a treatment are annotated on a graph, with (a,b,c) and significant differences within a line between treatments annotated with (x,y,z) (*p* ≤ 0.05). Reported correlation coefficients indicate significant correlation at *p* < 0.05.

## 3. Results

**Figure 1:**
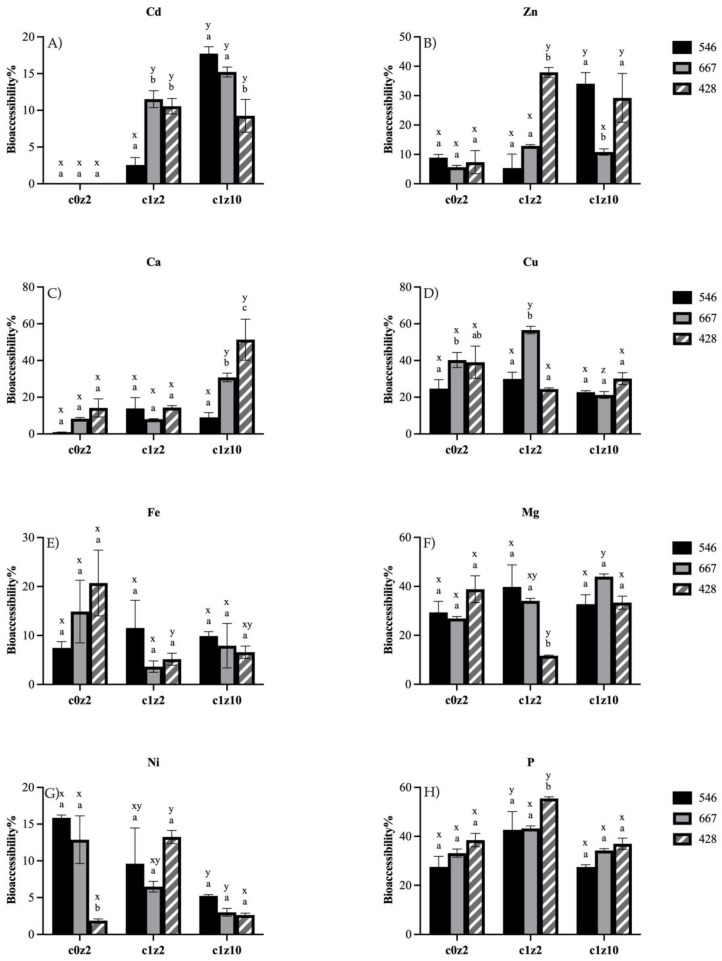
Mineral bioaccessibility (**A**–**H**) from grains of three rice cultivars grown in c0z2, c1z2, or c1z10. Bioaccessibility% is calculated as mineral content in soluble fraction/total grain mineral content in rice grains ×100. Values represent mean (*n* = 3) ± standard error. Different letters signify statistically significant difference (*p* < 0.05) between rice lines within a treatment (a,b,c) or between the three treatments within a rice line (x,y,z). Two-way ANOVA multiple comparisons with a Bonferroni correction was performed to analyze the differences.

The effect of Cd and Zn treatments on the bioaccessibility of cadmium and essential minerals was assessed in three lines of rice that differed in grain Cd concentration (Figure 1). The bioaccessibility of all minerals were influenced by line, treatment, or line x treatment.

Cadmium bioaccessibility (Figure 1A) varied significantly between the rice lines tested. In the c1z2 treatment, the low line (546) had significantly lower BA compared to the other two lines. However, in the Zn fertilization (c1z10) treatments, bioaccessibility increased significantly from 2.5% to 17.7%. The elevated Zn treatment (c1z10) did not, however, have a statistically significant effect on Cd BA in the high- (428) or medium (667)-grain Cd lines when compared to the c1z2 treatment. There was a slight decrease in the Cd BA (8.3%) in the high line when compared to the other two lines (19.5% in low and 15.3% in medium lines), when lines were grown with Cd and elevated Zn (c1z10).

There were no differences between the lines in Zn bioaccessibility under control (c0z2) treatments (Figure 1B), but significant differences existed between the lines at both c1z2 and c1z10 treatments. When Cd was present in either the c1z2 or c1z10 treatments, Zn BA in the high 428 line was almost 3x higher when compared to the controls. While high Zn fertilization did not cause a difference in Zn bioaccessibility in the high line (c1z2 vs. c1z10), the elevated Zn treatment with Cd (c1z2) did increase Zn bioaccessibility in the low 546 line. Neither Cd or Zn treatments had any effect on Zn BA in the medium 667 line.

For the other minerals tested (Ca, Cu, Fe, Mg, Ni, and P), significant differences between lines and treatments were present. There was no difference in Ca BA between the lines in the c0z2 and c1z2 treatments. However, Ca BA (Figure 1C) increased from 8.3 to 30.7% in the medium (667) line between the c1z2 and c1z10 treatments and from 14.1 to 51.3% in the high (428) line between the two treatments. For Cu, there were slight differences between the lines within each treatment. There were significant differences in BA in the medium 667 line between treatments. Copper BA (Figure 1D) was increased from 40 to 56% in the c1z2 treatment, but was reduced to 21% by the c1z10 treatment. It was interesting to note that Fe and Mg bioaccessibility decreased in the high (428) line (Figure 1E,F) from 20% to 5% and from 38 to 11%, respectively, in the c1z2 treatment, while the BA levels recovered for Mg in the c1z10 treatment. Nickel BA (Figure 1G) increased from 1.8 to 13.3% in the high line, but was lower in all lines in the c1z10 treatment. Phosphorous BA (Figure 1H) was increased by Cd treatments in both the low and high lines but was back to control levels in the Zn fertilization treatment (c1z10).

The effects of zinc fertilization on estimated Cd dietary exposure (DE) were calculated for men, women, and children separately. The dietary habits of consumers were estimated to be 100 g/day of grain consumed by 13 kg children, 320 g/day of grain consumed by 60 kg women, and 450 g/day of grain consumed by 70 kg men. Under c1z2 treatments, dietary exposure (Table 3) was lowest in the low Cd concentration line 546 and highest in the high Cd concentration line 428. Dietary exposure was highest for children reaching as high as 197.29 μg/kg/week from the high line (428). Dietary exposure rates always surpassed acceptable limits of 2.5 μg/kg body weight/week when these plants were grown in the presence of Cd. Alarmingly, Cd exposure from the medium (667)- and high (428)-Cd lines (grown under the conditions of this study) could lead to DE ranging from 53.99 to 197.29 μg/kg/week. The presence of 10 µM Zn in the growing media increased the Cd dietary exposure in both the low- and high-Cd lines but led to slight decreases in the medium line (667).

## 4. Discussion

Rice is a major source of Cd toxicity to humans causing severe health issues that are exacerbated by Zn malnutrition [59,60,61,62]. Research has focused on developing rice varieties that limit Cd and increase Zn but total concentrations in rice grains are not 100% bioaccessible to consumers [63,64,65]. Mineral bioaccessibility from foods is dependent on several factors including genotype, growing media, post-harvest processing, food cooking, mineral speciation, and a person’s nutritional status [43,66,67,68,69,70]. Interactions between Cd and Zn on the transport of minerals to rice grains have been reported on, but to our knowledge, the influence of Cd and Zn interactions on mineral bioaccessibility have not been explored. The goal of this work was to use an in vitro physiologically based extraction test to see if exogenous Cd and Zn affect the bioaccessibility of essential minerals and Cd in three different rice lines and to assess the potential risk from dietary exposure.

In our study, BA differed between the three lines, which is in agreement with previous studies that have shown that Cd BA can differ between cultivars [44,69,70,71]. Cadmium BA was significantly higher in the high and medium lines 667 and 428 but not in the low 546 line in c1z2 treatment. Unlike previous studies [58], our study showed that an increased grain Cd concentration does not always correlate to increased BA, as observed in the medium 667 and high 428 lines. One of the interesting findings from our study was that increasing Zn concentrations inadvertently increased Cd BA in the low Cd line (546). Attempts to increase Zn concentration in rice as a strategy to decrease Cd accumulation may unknowingly increase Cd BA instead of reducing BA, posing a health risk to consumers.

Mineral complexation in grains is one of the main factors influencing bioaccessibility of Cd, Zn, and other minerals. In rice grain, Cd is bound to glutathione, which is easily digested [72]. Zinc, and some other minerals such as iron, are often bound to phytate whose insolubility in the gastrointestinal tract limits its bioaccessibility and absorption [73]. Genetic control over the production of these secondary metabolites can be responsible for the variation in BA seen for essential minerals and Cd in our results [69,74,75,76].

As the bioaccessibility of Cd in rice increases, so does the risk of dietary exposure to consumers. Rice varieties that have a significant difference in Cd accumulation and bioaccessibility may be able to limit the risk of exposure. Tolerable limits of 2.5 μg/kg/week recommended by the World Health Organization [14,77] were quickly surpassed in all three rice lines tested here. Interestingly, Zn fertilization led to significant increases in the concentration, bioaccessibility, and exposure of Cd. This work is in agreement with a previous study [78], where adjusted dietary exposure for bioaccessibility suggested elevated risks for populations who consume rice varieties with high concentrations of Cd. Furthermore, our work suggests that Zn fertilization may exacerbate that risk, because Zn fertilization increased dietary exposure to Cd in both low and high lines. However, in the medium line, Zn fertilization seems to have slightly reduced dietary exposure values. Since rice is one of the highest contributors to dietary Cd, limiting rice Cd concentrations and bioaccessibility can help to reduce dietary exposure in children and adults [79,80].

While the physiologically based extraction test used here has limitations, it provides an inexpensive estimate by which the observations can be further expanded [81]. Future studies should use animal-model bioavailability studies to provide a more accurate health risk assessment from mineral interactions in rice. Health risk assessments would also benefit from analyzing mineral bioaccessibility and bioavailability in milled rice whose mineral content is usually lower than that of brown rice [82].

## 5. Conclusions

This work used an established physiologically based extraction test to analyze the effects of Cd and Zn interactions on the bioaccessibility of essential minerals and Cd from uncooked brown rice. Our findings show that exogenous Zn significantly increased Cd BA in a low grain Cd line of rice but did not affect Cd BA in a medium- or high-grain Cd rice line. Zinc BA, on the other hand, was significantly increased in the high-Cd line by Cd treatments or by both Cd and Zn treatments in the low-Cd line. The bioaccessibility of Ca, Cu, Fe, Mg, Ni, and P were also affected by growth treatments, suggesting growing conditions should be considered when fortifying plants to have higher mineral concentrations in edible tissues. The genotype was found to influence essential mineral and Cd bioaccessibility in these three lines of rice, which suggests low Cd bioaccessibility traits and mechanisms controlling these traits need to be identified for producing low-Cd foods. Future experiments analyzing the mechanisms that control genotypic differences in the bioaccessibility and bioavailability of Cd and essential minerals will be necessary to ensure the mitigation of health risks from consuming rice with low levels of essential nutrients or high levels of toxins like Cd. Together, this information can be used to reduce Cd and increase Zn absorption from rice.

## Figures and Tables

**Table 1:** List of rice lines. Rice was obtained from USDA Genetic Stock Oryza (GSOR) mini core [45]. Rice lines chosen represented diversity in grain Cd accumulation and geographic distribution. Line 310546 (546), Line 311667 (667), and Line 310428 (428) represent low-, medium-, and high-grain Cd accumulators, respectively.

GSOR	Experimental ID	Country of Origin
310546	546	Malaysia
311667	667	Mali
310428	428	Indonesia

**Table 2:** Mineral concentrations (mg/kg DW) in grains of rice lines 310546 (546/Low), 311667 (667/Medium), and 310428 (428/High) grown with a combination of different Cd and Zn concentrations: 0 μM Cd + 2 μM Zn (c0z2), 1 μM Cd + 2 μM Zn (c1z2), or 1 μM Cd + 10 μM Zn (c1z10) using CdCl_2_ and ZnSO_4_.

Treatment	Line	Ca	Cu	Fe	Mg	Ni	P	Zn	Cd
**c0z2**	546	1020.33	6.38	18.18	1358.99	1.71	2763.47	50.27	0.06
	667	103.01	7.71	15.28	1608.48	6.96	4022.68	33.45	0.03
	428	131.37	9.4	30.98	1212.23	10.19	3512.65	48.83	0.03
**c1z2**	546	140.62	5.76	14.67	1155.87	11.24	2588.96	41.84	0.3
	667	69.25	8.75	20.19	1357.87	8.2	3672.58	27.2	1.09
	428	229.96	11.12	55.56	1621.95	11.58	3633.39	52.92	2
**c1z10**	546	966.9	6.75	9.84	1417.82	2.49	3071.24	63.97	1.3
	667	77.95	7.38	14.8	1336.84	7.63	3781.59	45.23	0.82
	428	99.66	9.88	26.73	1556.22	14.46	2769.52	73.29	4.04

**Table 3:** Potential cadmium dietary exposure (DE) (µg/kg body weight/week) in children, women, and men if they consumed varieties of rice that were grown in 1 μM Cd + 2 μM Zn (c1z2) or 1 μM Cd + 10 μM Zn (c1z10).

	Line	Treatment	DE (μg/kg/week)
**Children**	Line 546	c1z2	4.81
		c1z10	126.81
	Line 667	c1z2	77.87
		c1z10	74.52
	Line 428	c1z2	121.07
		c1z10	197.29
**Women**	Line 546	c1z2	3.34
		c1z10	87.92
	Line 667	c1z2	53.99
		c1z10	51.67
	Line 428	c1z2	83.94
		c1z10	136.79
**Men**	Line 546	c1z2	3.85
		c1z10	101.50
	Line 667	c1z2	62.33
		c1z10	59.65
	Line 428	c1z2	96.91
		c1z10	157.92

## Data Availability

The data used to support the findings of this study can be made available by the corresponding author upon request.
